# Care of the human spirit and the role of dignity therapy: a systematic review of dignity therapy research

**DOI:** 10.1186/s12904-015-0007-1

**Published:** 2015-03-21

**Authors:** George Fitchett, Linda Emanuel, George Handzo, Lara Boyken, Diana J Wilkie

**Affiliations:** 1 Rush University Medical Center, 630 S. Hermitage Ave. Suite 505, Chicago, IL 60612 USA; 2 Buehler Center on Health, Aging & Society Northwestern University, 750N Lake Shore Dr. Suite 601, Chicago, IL 60611 USA; 3 HealthCare Chaplaincy Network, 65 Broadway, 12th Floor, New York, NY 10006 USA; 4 Center of Excellence for End-of-Life Transition Research, University of Illinois at Chicago College of Nursing, 845S. Damen Ave., M/C 802, Chicago, IL 60612 USA

**Keywords:** Dignity therapy, Literature review, Spiritual care, End-of-Life care

## Abstract

**Background:**

Dignity Therapy (DT), an intervention for people facing serious illness, focuses on dignity conservation tasks such as settling relationships, sharing words of love, and preparing a legacy document for loved ones. Research on DT began more than a decade ago and has been conducted in 7 countries, but a systematic review of DT research has not been published.

**Methods:**

Using a PubMed search with key terms of ‘dignity therapy’, ‘dignity psychotherapy’, ‘Chochinov’, and ‘dignity care’, we found 29 articles on DT and retained 25 after full-text review.

**Results:**

Of these, 17 articles representing 12 quantitative studies establish that patients who receive DT report high satisfaction and benefits for themselves and their families, including increased sense of meaning and purpose. The effects of DT on physical or emotional symptoms, however, were inconsistent.

**Conclusions:**

Conclusions point to three areas for future research on DT, to determine: (1) whether the DT intervention exerts an impact at a spiritual level and/or as a life completion task; (2) how DT should be implemented in real world settings; and (3) if DT has an effect on the illness experience within the context of not only the patient, but also the family and community. Building on this body of DT research, investigators will need to continue to be sensitive as they involve participants in DT studies and innovations to facilitate the generation and delivery of legacy documents to participants near the end of life.

## Background

Care of the psychological and spiritual aspects of a person during illness are recognized as essential components of patient-centered care. That the achievement of well-being in the face of incurable illness depends on these aspects of a person’s experience is particularly well acknowledged [1,2]. Research addressing social and psychological needs during physical illness has become more substantial in recent decades [1,3]. However, systematically developed, manualized, and well-studied interventions for these dimensions lag behind those for physical aspects of illness [4,5]. This paucity is even more pronounced for spiritual care than it is in psychological and social care, despite growing evidence for its importance [6-13].

Chochinov proposed Dignity Therapy (DT) as a psychotherapeutic intervention for people facing serious illness [14]. DT focuses on dignity conservation tasks such as settling relationships, sharing words of love, and preparing legacies of memory and shared values, all of which take on a heightened importance at the end of life. DT has some similarities with Butler’s Life Review [15], which Butler understood as part of a life-cycle task and developed as an antidote to depression in older adults. Both DT and Life Review are conceptualized as multi-dimensional psychosocial interventions for patient-centered care [14,15]. Perceiving that dignity depends on experiences of generativity and the pursuit of purpose and meaning, Chochinov [16] identified aspects of dignity-conserving care and proposed a model for its development, study, and use by clinicians to promote maintenance of dignity for patients facing serious illness [16]. This model includes spiritual as well as psychosocial and physical elements.

Dr. Chochinov and his colleagues developed a manualized guide for DT [17]. The intervention uses 10 core questions that guide an interview, including “*What are your most important accomplishments, and what do you feel most proud of? What are your hopes and dreams for your loved ones? What have you learned about life that you would want to pass along to others?”* [17].

Responses are used to create a written legacy document that the person shares with others important to him or her. Dr. Chochinov and his team provide standardized training for DT on an annual basis. Professionals from a range of disciplines deemed to have suitable prior mental health-related training (e.g., psychologists, social workers, chaplains, or physicians) from locations across the globe attend this training. In their manual, the steps of the DT intervention are described in detail to allow intervention fidelity. During consent and introduction to the DT process, the interventionist describes DT as an opportunity to speak about issues that he or she believes are most important and would want preserved as part of a legacy-making exercise. The DT session is audio-recorded, transcribed, edited, and given back to the patient, who may give it to family members or friends. The editing process gives the patient the freedom during the interview to share his or her free-form thoughts, knowing that there will be a step to reorganize and rework the transcript to produce a legacy document that he or she feels is aesthetically pleasing, accurate in content, and not damaging to anyone. The patient reviews the edited document and can then give it to family and friends.

A number of studies of DT have been conducted over the past decade. Multiple studies describe widespread acceptability and high satisfaction among those who experience DT. In contrast, rigorous evidence for any beneficial effects of DT on important palliative care outcomes has been limited. This contrast raised our curiosity about how measured outcomes related to the mechanism and timeline of DT’s impact; during this inquiry, we noted that research on DT has not been systematically reviewed. The purpose of this article is to provide a synthesis of findings from existing DT studies regarding its feasibility, acceptability, and effects, and from that synthesis, to propose next steps for further DT-related research.

## Methods

### Search strategy

**Figure 1:**
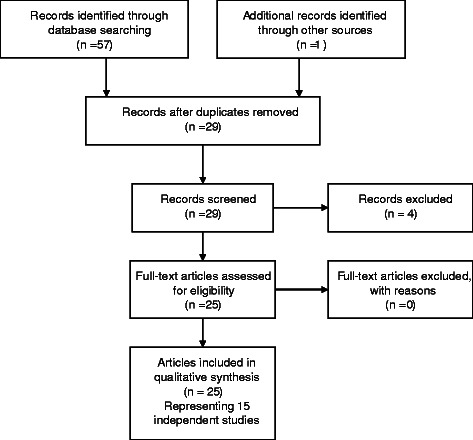
**Flow diagram of systematic review process.**

We queried the PubMed database to obtain an initial list of potential articles for review. As development of DT began in the 2000s, our searches included articles published in that decade through July 2014. The initial search terms were ‘dignity therapy’, ‘dignity psychotherapy’, ‘Chochinov’, and ‘dignity care’. As indicated in Figure 1, following PRISMA guidelines for systematic review [18] we identified and downloaded 57 references into EndNote X4 (Thompson Reuters ISI ResearchSoft, 2014). We deleted duplicate articles and retained 28 articles. An additional *in press* study [19] that was known to us (Linda Emanuel is a co-author) was added to the references, for a total of 29 articles.

### Selection criteria

The authors conducted a two-stage review, first of abstracts and then of full text articles. All authors reviewed both abstracts and articles from the review. We retained only studies of DT as an intervention. The retained 25 articles included qualitative and quantitative studies of the feasibility, acceptability, and effects of DT for patients (Figure 1). We excluded studies that focused on the development of, or other application of, the dignity therapy model (e.g., Hall et al. [20] Johnston et al. [21], Li et al. [22]), studies of DT and the family (rather than the patient), and other dignity or palliative care topics.

## Results

Our search yielded 17 articles that represent 12 quantitative studies of DT, which were led by principal investigators who conducted the studies: during their research training (3 studies); were experienced investigators (7 studies); or were of unknown prior investigator experience (2 studies). There were 8 uncontrolled feasibility studies and 4 randomized clinical trials (RCTs) for efficacy (Table 1) [17,19,23-38]. There were also 5 qualitative studies of DT, 4 of which occurred in a sample already involved in one of the quantitative studies (Table 2) [34,39-42]. Lastly, 3 case reports of DT, including 2 in patients with mental illness not at the end of life, and one in patients involved in one of the quantitative studies, were found (Table 3) [34,43,44]. In total, we reviewed 25 articles representing 15 separate studies.

**Table 1:** **Dignity therapy studies**

Study	Sample	Design	Measures	Findings
**Feasibility studies**				
Passik et al., 2004 [23]	US sample; 8 pts with cancer in hospice care	Single group pre-post trial of DT via telemedicine	• ZSDS	DT is feasible by telemedicine/ videoconference.
			• PUBs	
			• Single item scale for depression	
		Assessments: Baseline & immediate post DT		
			Evaluation	
			Satisfaction survey	
Chochinov et al., 2005 [17]	Canada and Australia sample	Single group, pre-post trial of DT	Single item screening measures for:	Significant improvement in: suffering (p = .023), depressive symptoms (p = .05).
			• Depression	
	100 terminally ill pts in hospital, NH or home	Assessments: Baseline & immediate post DT	• Dignity	
			• Anxiety	High proportions gave positive evaluation for benefits of DT: 91% reported feeling satisfied/ highly satisfied with DT.
			• Suffering	
			• Hopefulness	
			• Desire for death	
				86% reported DT was helpful or very helpful.
			• Suicide	
			• Sense of well-being	81% indicated DT had already helped, or would help, their family.
			QoL – 2 items	
			ESAS (revised)	76% indicated DT heightened their sense of dignity.
			*Evaluation*	
			DTPFQ	47% indicated DT increased their will to live.
Akechi et al., 2012 [24]	Japanese sample;	Two trials of DT feasibility, no control	*Evaluation*	Major problems with recruitment in Study 1 but not Study 2. The authors raise concern about acceptability of DT in Japanese culture.
			DTPFQ– 9 items	
	11 pts with advanced cancer in hospice and hospital pall care unit	Assessment: No schedule of assessment reported		
				Overall positive evaluation of DT: 67% indicated DT heightened their sense of dignity, 56% indicated DT was beneficial, 78% indicated DT had already helped, or would help, their family.
Chochinov et al., 2012 [25]	Canadian sample;	Single group trial of DT	*Evaluation*	Cognitively intact: reports helpful and satisfactory, but no specification of benefits around meaning, purpose, dignity.
			DTPFQ– 9 items	
		Assessment: 2–3 days post DT		
	12 cognitively intact and 11 cognitively impaired frail elderly in long-term care			
				Cognitively impaired: proxy participants (family members) indicated DT is helpful to them and their families.
Johns 2013 [26]	US sample; 10 pts with metastatic cancer from community or outpatient oncology unit	Single group trial of DT	• 7-item cancer distress measure	4 completers (3 declined to finish, 3 deaths); sample size too small for statistical analyses.
			• BDI	
		Assessment: Baseline & f/u 1 shortly post DT, f/u 2 within 1 month	• FACIT-PAL	
			*Evaluation*	Feasibility and acceptability reported from surviving participants.
			DTPFQ	
Bentley et al., 2014 [27,28]	Australian sample;	Single group pre-post trial of DT	• PDI	Feasibility and acceptability established.
			• FACIT-SP	High rating of satisfaction (93%) and helpfulness (89%) for DT.
	29 pts diagnosed with motor neurone disease living in community	Assessment:	*Evaluation*	
		Baseline, f/u	• DTPFQ	
		1 wk post DT		
			• 3 additional single-item measures of hopefulness & family support.	No significant changes in hope, spirituality or dignity.
Houmann et al., 2014 [29,30]	Danish sample; 80 pts w incurable ca from hospice and hospital pall care unit	Single group pre-post trial of DT	• SISC - 6 items	No change on any measure at f/u 1 or f/u 2 except QoL decreased baseline to f/u 1.
			• PDI	
			• Quality of life: EORTC QLQ-C15-	
		Assessment:		At f/u 1 and f/u 2 positive responses on DTPFQ.
		Baseline, f/u 1 immediately after receiving generativity document (median 36 days post DT), f/u 2 1 mo after DT (median 60 days)	• PAL	
			• HADS	Issues w recruitment (~50%)
			• Palliative	Issues w retention f/u 1 69%, f/u 2 39%.
			• Performance scale ver2 (PPSv2)	
			*Evaluation*	Issues with floor effects (30%-70% no sx on SISC/PDI).
			DTPFQ– 9 items	
				Subgroup analysis for each SISC/PDI item using those with some sx found some improvement for selected items.
Vergo et al., 2014 [19]	US sample;	Single group pre-post trial of DT	• TIA	DTPFQ indicates DT very acceptable; increase in death acceptance over time (11% at baseline vs. 57% at f/u 2).
	15 pts with stage-IV colorectal cancer receiving palliative chemotherapy		• Distress Thermometer	
		Assessment:		
			• ESAS	
		Baseline, f/u 1	• 2-item QOL	
		2–3 weeks post-DT, f/u 2 1 month post-DT	• H-CAP-S	
			*Evaluation*	
			DTPFQ – 5 items	
**Efficacy studies**				
Chochinov et al., 2011 [31]	Canada, Australian, & US sample	3 arm RCT: DT vs client-centered care vs standard care	Outcomes	No significant differences on any of the outcomes.
			• Structured Interview for Symptoms and Concerns (SISC): 7 items	
				Pts in the DT group had higher scores than the other grps on 8 of 22 evaluation items.
	441 pts receiving palliative care in hospital, hospice or home			
		Assessment:		
			• Edmonton Sx – 8 items	Issues with recruitment, retention, floor effects.
			• Quality of life: 2 items	
		Baseline & f/u immediately post receiving generativity document	• HADS	
			• FACIT-Sp	
			PDI	
			*Evaluation*	
			DTPFQ– 22 items	
		*Intervention*: psychiatrist, psychologist, palliative care nurses		
Hall et al., 2011 [32,33,36]	UK sample	Design RCT (Phase II trial for acceptability, estimates of effect sizes): Tx = DT plus usual care; Control = usual care	Primary:	No differences on PDI.
			• PDI	
				No differences for any secondary outcomes, except higher hope in DT grp at f/u 1 (p = .02).
	45 pts with advanced cancer		Secondary:	
			• Hope	
			• HADS	
			• EQ-5D	Patients in the DT group had higher scores on DTPFQ, some significant.
		Assessment:	• palliative-related outcomes (Hearn)	
			DTPFQ	
		Baseline, f/u 1 1 week post DT, f/u 2 4 wks post-DT.		
		*Intervention*: oncologist		
Hall et al., 2012 [35,36]	UK sample	Design RCT (Phase II trial for potential effectiveness, feasibility): Tx = DT plus usual care; Control = usual care	Primary:	No significant differences on effectiveness measures at any point; reduced dignity-related distress as measured by DTPFQ across both groups (p = 0.026).
	64 pts in older care homes		• PDI	
			Secondary:	
			• GDS	
			• HHI	
			• EQ-5D	
			Acceptability:	Patients in the DT group significantly more likely to feel DT had made life more meaningful at f/u 1 (p = 0.04).
			• DTPFQ	
		Assessment:		
		Baseline, f/u 1 7 days post-DT, f/u 2 8 wks post-DT		
		*Intervention*: palliative care nurse		
Juliao et al., 2014 [37,38]	Portuguese sample	Design RCT: Tx = DT+ usual care; Control = usual care	HADS	DT associated with lower depression at f/u 1 and 2 (p < 0.0001) and lower anxiety at f/u 1, 2 and 3 (p < 0.0001).
	60 terminally ill pts			
		Assessment:		
		Baseline, f/u 1 4 days post-DT, f/u 2 15 days post-DT, f/u 3 30 days post-DT		
		*Intervention*: palliative care physician		

BDI = Beck Depression Index; DTPFQ = DT Patient Feedback Questionnaire; EORTCQLQ-C15-PAL = European Organization for Research in Cancer Quality of Life Questionnaire; EQ-5D = EuroQol; ESAS = Edmonton System Assessment Scale; FACIT-SP = Functional Assessment of Chronic Illness Therapy - Spiritual Well-Being; FACIT-PAL = Functional Assessment of Chronic Illness Therapy – Palliative Care; GDS = Geriatric Depression Scale; HADS = Hospital Anxiety Depression Scale; H-CAP-S = Hypothetical Advanced Care Planning Scenario; HHI = Herth Hope Index; NH = nursing home; PDI = Personal Dignity Inventory; PPSv2 = Palliative Performance Scale; PUBs = Purposelessness, Understimulation and Boredom scale; QoL – 2 = Quality of Life; SISC = Structured Interview for Symptoms and Concerns; TIA = Terminal Illness Acknowledgement; ZSDS = Zung Self-Rating Depression Scale.

**Table 2:** **Qualitative studies of dignity therapy**

Study	Sample	Methods	Main study finding
Hack et al., 2010 [39]	50 edited DT transcripts (17 Canadian and 33 Australian) from patients with terminal illness in inpatient palliative care programs, sample from Chochinov et al., 2005 [17]	Content analysis, constant comparative analysis of completed DT legacy document by three investigators	• Throughout DT interview patients reflect on two to three personally meaningful core values, such as ‘family’, ‘pleasure’, ‘caring’, and ‘sense of accomplishment’.
			• DT is used by patients to confirm personal identity.
			• Investigators suggest more theoretical analysis of “meaning-making” construct in end-of-life care needed.
Tait et al., 2011 [40]	12 Canadian patients with terminal illness in inpatient palliative care	Constant comparative analysis of DT interviews	• Three main ‘types of interviews’ emerge: ‘Evaluation narratives’, focusing on life prior to illness; ‘transition narratives’, focusing on change in health status and its meaning; ‘legacy narratives’, focusing on future without the patient.
			• Investigators suggest narrative themes share commonality with medical interview and eulogy genres.
Montross et al., 2011 [41]	27 US community-based hospice patients	Coding consensus, co-occurrence, and comparison analysis of DT legacy documents	• Similar findings to Hack et al., 2010 [39], core values consistently expressed in transcripts.
			• DT is feasible in a community-based setting.
Hall et al., 2013 [34]	49 UK pts in older care homes, sample from Hall et al., 2012 [35,36]	Framework analysis of qualitative interviews conducted at T1 and T2; interviews on resident views of DT and/or being a study participant (control group).	• Of 9 themes, 3 were unique to intervention group: ‘views of legacy document’; ‘generativity’; and ‘reminiscence’.
			• DT not recommended by investigators, in current form, with participants with cognitive impairment: findings suggest DT document may reflect ‘distorted sense of self’ and prompt distress.
Hall et al., 2013 [42]	29 UK pts with advanced cancer, sample from Hall et al., 2011 [32,33]	Framework analysis of qualitative interviews conducted at T1 and T2; interviews on pt views of DT and/or being a study participant (control group).	• 5 of 7 themes in Dignity Model theory present in both interviews groups; ‘generativity’ found only in intervention group.
			• No evidence of ‘role preservation’ as described in Dignity Model in this sample.
			• Qualitative interview reporting of higher levels of hopefulness in both groups from participating in study, despite no change in quantitative component of study.

**Table 3:** **Case reports of dignity therapy use**

Study	Sample	Implementation	Discussion
Avery & Savitz, 2011 [44]	US patient with schizoaffective disorder in inpatient psychiatric unit	DT protocol questions used by patient to write life story, prompted by worries of not spending time with family because of illness. Investigator typed and edited narrative and discussed with patient.	• Patient reported that narrative had ‘restored hope’ to him. Patient shared copies of document with loved ones.
			• Investigators note DT could be beneficial for pts with chronic mental illness, “improving patient narratives”.
Avery & Baez, 2012 [43]	US patient with major depressive disorder in inpatient setting	DT protocol used by investigator to aid patient in ‘gaining fresh perspective’ after severe depression following loss of job.	• Patient reported DT aided her in ‘finding hope’, and improved her mood.
			• Investigator notes use of DT to make sense of major life event and loss.
			• Investigator posits DT legacy document may be supportive to family members of patients with chronic mental illness.
Hall et al., 2013 [34]	3 UK patients with advanced cancer in high distress, sample from Hall et al., 2011 [32,33]	Focus on ‘dignity-related problems’ expressed by patients, qualitative review of DT legacy documents.	• Investigators note DT administered in a context of complex and quickly changing circumstances.
			• Distressed patients may find focus on ‘overarching truths, feelings and insights’ as indicated in DT protocol very difficult.
			• DT therapeutic relationship is challenging with patients who are distressed given short time-frame of interaction.

### Settings

The investigators of the quantitative studies recruited samples from Canada, Australia, the United States, the United Kingdom, Denmark, Portugal, and Japan. Their sample sizes ranged from 8 to 441. Participants in these studies were primarily people who were terminally ill or had advanced cancer, and were receiving care either at home, in a nursing home, or in the hospice or palliative care units of a hospital. There were 2studies whose participants were older adults living in long-term care settings.

### Design

The quantitative studies included 8 with a pre/posttest single group design, 3 with a two-group RCT design, and one with a three-group RCT design. Only one RCT study [31] was adequately powered to test efficacy of the DT intervention (Table 1). All but one study included a baseline measurement before the DT intervention. Studies included 1 or 2 measurements after the intervention at intervals that varied substantially, from immediately post-intervention (3 studies) to 2 months post-intervention. These posttest measures typically occurred within a week of the intervention (6 studies), but also occurred 2 to 4 weeks after the intervention (4 studies), and 2 months after the intervention (1 study).

### Intervention

Investigators used Chochinov’s DT protocol in all 12 quantitative studies. In one study, based on participant preference, the investigator expanded the legacy document to include photographs, and delivered the legacy book at a later time after the intervention than the other studies [26]. Chochinov trained the DT therapists for 8 studies. The type of training was not specified for 3 studies [23,24,38], and in one study, the investigator independently followed the published literature on DT [26].

### Measures/approaches

In the quantitative studies, investigators used a variety of measures to examine primary and secondary outcomes. These included measures of: depression (9 studies), anxiety (6 studies), symptoms (4 studies), quality of life (7 studies), hope (5 studies), spiritual well-being (2 studies), dignity (7 studies), function (3 studies) and other psychosocial or palliative care-related concepts (5 studies). The tools for the designated primary outcome in the efficacy studies were the Personal Dignity Inventory (2 studies), Hospital Anxiety Depression Scale (1 study), and other tools (1 study). The tools for the secondary outcomes varied among the efficacy studies as listed in Table 1. The DT Patient Feedback Questionnaire was used in most of the quantitative studies either as a measure of acceptability or as an outcome measure. The investigators of the 5 qualitative studies used content analysis with coding consensus as the main analytic technique. Additional techniques included constant comparative analysis, framework analysis, and co-occurrence analysis. The case studies described novel cases of distressed patients.

Based on the existing quantitative and qualitative studies, we summarize key findings regarding the acceptability, feasibility, and effects of DT. Effects include primary and secondary outcome findings.

### Acceptability

Evidence of DT’s acceptability is clear and consistent; patients who receive DT provide exceptionally high ratings of satisfaction and benefits for themselves and their families. For example, in a study of 100 terminally ill patients in Canada and Australia who received DT, Chochinov and colleagues reported that 91% reported feeling satisfied or highly satisfied with DT, 76% indicated that DT heightened their sense of dignity, and 47% reported that DT increased their will to live [17]. Similarly, among 29 Australian patients diagnosed with motor neuron disease who received DT, 93% provided high ratings of satisfaction, and 89% reported high ratings of helpfulness [28]. Among the participants in the study by Chochinov and colleagues, 81% indicated that DT had already helped, or would help, their family [17].

### Feasibility

Recruitment and retention were issues for almost all of the DT studies and a major problem for some. For example, in a Danish study of patients with incurable cancer, only 21% of potential participants were eligible, it took two years to recruit a sample of 80 patients, and only 31 patients (39%) completed the second follow-up assessment [30]. In an RCT of DT among 45 patients in the UK with advanced cancer, the recruitment rate was 24%, 60% of the participants completed the 1-week follow-up assessment, and only 44% completed the 1-month follow-up [33]. In an RCT of DT among 60 terminally ill Portuguese patients, only 30 (50%) were available for the 30-day follow-up assessment [37]. Problems with recruitment and retention are not unexpected in research with persons with advanced illness receiving palliative care [45] and they clearly affected these studies of DT.

### Effectiveness

In single group studies, DT often showed significant changes in study outcomes. When compared to usual care control groups, however, DT effects were often not statistically significant. Only one study [31] was powered to detect small or moderate effects in study outcomes (sample size = 441, divided into 3 groups). In that study, compared to participants in the other study arms, there were no significant differences for any primary outcomes for participants who received DT. The other 3 RCTs were much smaller (n = 45, 60, or 64) and may have been underpowered [33,36,37]. However, in one of those studies, compared to usual care controls, participants who received DT had significantly lower levels of anxiety and depression at follow-up [37]. In the 3 RCTs that examined satisfaction with DT, compared to those who did not received DT, participants who received DT reported higher levels of meaning in life, quality of life, and spiritual well-being post-intervention [31,33,36].

## Discussion

In contrast to the strong evidence of the acceptability of DT, the existing studies raise several questions about DT’s feasibility and efficacy. We take up these questions below.

### The nature of DT and how to measure what it does

In the largest, most definitive study of DT to date, the absence of effects on many outcomes immediately after DT is striking, especially in view of how positively participants report their response to DT. This conclusion is critical. From the scientific evidence, we cannot yet say if DT is efficacious, what it is efficacious for in the short term, or why reported effects emerge later in a single group study. This may be an issue of what is being measured. One possible explanation, noted by Hall and colleagues [33], is that DT does not directly address physical symptoms or functions, and thus it is a mistake to use as primary outcomes measures that include them, such as those in the Personal Dignity Inventory.

The possible need to use delayed measurement is raised by Vergo and colleagues’ small (n = 15; 88% participation) feasibility study of DT among outpatients with stage IV colorectal cancer actively receiving second line chemotherapy [19]. Because the investigators were interested to know if DT improves a person’s existential maturity (comfort with their mortality) [46], outcomes included death acceptance (peaceful awareness and treatment preferences). Although the study was small, its findings suggest DT may contribute to increased understanding of the terminal nature of their disease, and less aggressive end-of-life goals of care. Specifically, there appeared to be an increase in death acceptance over time (11% at baseline and 57% at 1 month post-DT), raising the possibility of a delayed effect, which is consistent with findings from other studies of group differences in effects that occurred a week or more after DT [31,37].

While acknowledging that DT is designed to work in multiple dimensions, we are particularly interested in its action in the spiritual dimension of peoples’ lives. DT’s conceptual framework includes a spiritual component, and recent work indicates a taxonomy of chaplaincy activities reasonably aligned with most DT components [47-49]. As people age, many become more spiritually inclined as a life-cycle phenomenon [50,51]. A substantial body of evidence describes the relationship between religious involvement and beliefs and preferences for end-of-life care, as well as the care actually received; generally, higher levels of religious involvement are associated with preferences for and receipt of life-prolonging treatment [52-55]. While this association has been observed in multiple studies, the reasons for it are less well-understood. One possibility is that unfinished spiritual or religious tasks led to preferences for life-prolonging care at the end-of-life [56]. We propose that DT should be studied as a spiritual as well as a psychosocial intervention that assists with the existential tasks faced by the majority of elderly patients, especially as they encounter a life threatening illness like cancer. Our conceptualization of DT as an intervention with a strong spiritual element leads us to note that, while they have not been used in prior studies, measures of the existential tasks associated with the end of life, as well as dignity impact, are appropriate outcome measures for studies of DT. For instance, studies to further investigate the preliminary findings by Vergo and colleagues on DT’s impact on illness acceptance and care goals will be important.

We also note that, as described in the model for understanding care of the human spirit (in this issue), the mechanism of action of a spiritual intervention might have unique features, such as its timing and interactions with the other main spheres of human experience. Therefore, careful thought about what might mediate DT outcomes is warranted. For instance, pain, depression, or role incompletion might mediate outcomes, perhaps limiting desired and amplifying undesired states. Or if, as the model hypothesizes, there is a reciprocal interaction between spheres, this might explain the impact of DT, which might act through multiple spheres to have a primary spiritual impact. Further understanding will require creative approaches, perhaps even involving bundled interventions, and a reconsideration of outcome measures.

Some of these issues will be challenging to resolve because of the **feasibility of conducting studies of DT**. As has been noted [45], recruitment and retention is challenging in seriously ill populations. If, in determining the ideal interval between the completion of DT and follow-up assessment of outcomes, it appears that more time is needed – either to allow for changes that may emerge over time as the participant reflects on the DT experience, or as changes occur that may result from the participant sharing his or her legacy document with significant others – the retention issue may be exacerbated. That is, both of these potential reasons suggest a longer interval is needed before follow-up assessment, at least one week, and perhaps longer. However, the tradeoff is that longer intervals are associated with serious attrition due to deteriorating health or death.

Related to issues of recruitment and retention is the fact that patients who are experiencing higher levels of distress may be less likely to volunteer to participate in DT studies, or to complete them. This appears to have been the case in some studies of DT where the investigators noted the low levels of distress in the samples they recruited [31,33,36]. This results in ceiling and floor effects in the measures of pre-intervention distress that preclude an intervention such as DT from having any measurable effect on those outcomes [57,58]. One solution is to screen and select study participants for elevated pre-intervention distress, a choice that would likely exacerbate issues with recruitment.

Aside from issues of feasibility of DT research, there is emerging evidence about the **feasibility of DT in routine clinical practice**. In their study of DT with 27 patients in a community-based hospice, Montross and colleagues [41] reported that the mean number of sessions for completion of DT was 4, with an average of 6.3 hours devoted to DT per patient. This estimate did not include the time required for editing the DT legacy documents (see Hall et al.’s report [36] of personal communication with Montross). In the Montross et al. study, the average length of the legacy documents was 8 single-space pages, which, at a transcription cost of 13 cents per line of text, yielded an average transcription cost of $56 (range $27 to $144) per document. This team concluded these costs were “reasonable and at a level that is readily sustainable for the organization” (p. 733 [41]). In contrast, in their study of DT with older adults living in care homes, Hall and colleagues [36] reported an average of 4 hours for transcription of the legacy documents and a total time for DT of 15.04 hours (SD = 7.13), which included time for the DT interview, transcription, and returning and revising the legacy document. In this study, for 30% of the participants, 4–6 visits were required to review and revise the legacy document, in part because in subsequent visits participants recalled additional information they felt should be included.

A remaining clinical feasibility question relates to **who should administer DT** in a real-life care setting. A reasonable approach to this question would be to conceptualize the dignity therapist either as a generalist (physicians, nurses) or a specialist (psychologists, chaplains). Within palliative care teams, it is most likely that nurses or chaplains could absorb DT as part of their routine work. In a prior RCT [31], a research nurse administered the DT, with significant efficacy findings. Further, the nursing discipline’s focus on holistic care, inclusive of spirituality, makes this discipline a strong candidate [21]. Johnston et al. [21], recommended a nursing intervention derived from the Chochinov dignity model – the dignity care pathway – to assist nurses in conserving patient dignity at the end of life. However, although the ratio of nurses to patients is suitable, nurses already have a heavy workflow that may be sidetracked by DT unless carefully scheduled.

Recent findings [56] also indicate that clinicians from nursing and medicine look to board certified chaplains as the health care professionals with the expertise to provide spiritual care [9]. Chaplain-to-patient ratios and chaplain assignments are not currently suitable for the routine offering of DT, but chaplains might be more interested in DT than nurses due to significant and growing demand for evidence-informed interventions for chaplains. Studies are needed to evaluate DT efficacy by different health care professionals and in diverse settings. For instance, where the therapy should be done, and when in the sequence of the patient’s clinical encounters, who should transcribe the interview, how to return the legacy document, and how long an interval between transcription and delivery is suitable, etc. are not established for clinical practice. The impact on the flow of the team’s work, and the chaplain or nurse in particular, is not known.

### Who should receive DT?

Although our review focused solely on uses of DT as an intervention for patients, a small body of literature is developing in which investigators are studying the effects of DT on family members of patients who have participated in DT. As noted in Table 1, Chochinov et al. [25] present a novel use of DT by proxy family members of patients with cognitive impairment. The majority of family members involved indicated they would recommend it to other long-term care residents and their families. Goddard et al. [59] report similar findings of family member recommendations for DT in a long-term care setting. In a study for family caregivers of those with motor neuron disease [60], the investigators report that family caregivers identified benefits to the patient participating in DT, and to themselves during bereavement, but they present mixed findings about the acceptability of the intervention to family members at the time of the intervention. The investigators recommend that dignity therapists be sensitive to acceptability issues and the quality of patient and family caregiver relationships. Other preliminary investigations into the receptivity and acceptance of DT by family members [61], [26] and hospice staff [62] are reported in the literature. Together, these studies present rich but preliminary evidence that DT has positive effects not only on the patients who receive it, but on their families and caregivers. In addition to mechanism and efficacy studies of DT, more research is needed to enhance understanding of the best practices in implementing DT in consideration of family members and others involved in patient care.

## Conclusion

Our literature review finds robust evidence for DT’s overwhelming acceptability, rare for any medical intervention, especially in psycho-social-spiritual care. This evidence exists despite challenges to the feasibility of DT because of recruitment and retention difficulties among very ill populations. Potentially exacerbating this challenge for future studies, it remains possible that measures for DT should occur a week or more after the intervention to capture its impact. The evidence for DT’s immediate efficacy is lacking in the only fully powered RCT so far available, in which significant impact was limited to secondary outcomes only measured post-intervention. However, smaller studies did find effects for increased hopefulness [32,33,36] and lower depression and anxiety [37,38]. DT was designed based on an empirically derived model and includes elements from several domains of the human experience; its measures have reflected this. Nonetheless, available evidence suggests that the mechanism of DT action may not be related in a straightforward way to its underlying model. In particular, we perceive that its mechanisms may be related to role completion and spiritual aspects of a person’s life. In addition, and relatedly, we note that clinical feasibility studies are needed for DT. It is important to establish how best to implement such a well-received intervention into clinical practice. We suggest that future research on DT should focus on three main areas. The first pertains to the nature of the intervention, especially the possibility that its main impact is in a spiritual dimension. The second relates to how DT should be implemented in real world settings. The third relates to the reality that illness is experienced not only by the patient but by the family and community.
